# Duration of Chemotherapy-induced Nausea and Vomiting (CINV) as a Predictor of Recurrent CINV in Later Cycles

**DOI:** 10.1093/oncolo/oyac240

**Published:** 2022-12-17

**Authors:** Rudolph Navari, Gary Binder, Alex Molasiotis, Jørn Herrstedt, Eric J Roeland, Kathryn J Ruddy, Thomas W LeBlanc, Dwight D Kloth, Kelsey A Klute, Eros Papademetriou, Luke Schmerold, Lee Schwartzberg

**Affiliations:** Cancer Care Program, World Health Organization, Geneva, Switzerland; Helsinn Therapeutics US Inc., Iselin, NJ, USA (currently Servier Pharmaceuticals); College of Arts, Humanities & Education, University of Derby, Derby, UK; Department of Clinical Medicine, Faculty of Health and Medical Sciences, University of Copenhagen, Copenhagen, Denmark; Department of Clinical Oncology, Zealand University Hospital Roskilde, Denmark; Oregon Health and Sciences Center, Knight Cancer Institute, Portland, OR, USA; Department of Oncology, Mayo Clinic, Rochester, MN, USA; Division of Hematologic Malignancies and Cellular Therapy, Department of Medicine, Duke Cancer Institute, Durham, NC, USA; Department of Pharmacy, Fox Chase Cancer Center, Philadelphia, PA, USA; University of Nebraska Medical Center, Buffett Cancer Center, Omaha, NE, USA; SmartAnalyst, Inc., New York, NY, USA; SmartAnalyst, Inc., New York, NY, USA; Renown Institute for Cancer, Reno, NV, USA

**Keywords:** breast cancer, nausea, vomiting, chemotherapy, antiemetic

## Abstract

**Background:**

The relationship between CINV duration and recurrence in subsequent cycles is largely unstudied. Our objective was to determine if patients experiencing CINV in their first cycle of chemotherapy (C1) would face increased risk of CINV in later cycles and whether the duration of the CINV would predict increased risk of recurrence.

**Patients and Methods:**

Using data from a previously reported phase III trial, we assessed patients’ recurrence of breakthrough CINV after antiemetic prophylaxis for anthracycline+cyclophosphamide (AC) for breast cancer, comparing C1 short CINV vs. extended CINV as a secondary analysis. Complete response (CR) and CINV duration were primary and secondary endpoints, respectively. CR was considered prophylaxis success; lack of CR was considered treatment failure (TF).

**Results:**

Among 402 female patients, 99 (24.6%) had TF in C1 (TF1). The remaining 303 patients (CR1) had ≥93% CR rates in each subsequent cycle, while the 99 patients with TF1 had TF rates of 49.8% for cycles 2-4 (*P* < .001). The 51 patients with extended TF (≥3 days) in C1 had recurrent TF in 73/105 later cycles (69.5%, *P* < .001), while the 48 patients with short TF (1-2 days) in C1 had recurrent TF in 33/108 later cycles (30.6%). The relative risk of recurrence after C1 extended TF was 2.28 (CI 1.67-3.11; *P* < .001) compared to short TF.

**Conclusions:**

Prophylaxis success in C1 led to >90% repeat success across cycles of AC-based chemotherapy. For patients with breakthrough CINV, extended duration strongly predicted recurrent CINV. The duration of CINV should be closely monitored, and augmenting antiemetic prophylaxis considered for future cycles when extended CINV occurs.

## Teaser

The relationship between the duration and recurrence of chemotherapy-induced nausea and vomiting (CINV) is largely unstudied. This article reports on whether patients experiencing CINV in their first cycle of chemotherapy have increased risk of CINV in later cycles and whether the duration of the CINV could predict increased risk of recurrence.

**Implications for Practice**

Chemotherapy-induced nausea/vomiting that occurs for more than 2 days in the first cycle of highly emetogenic chemotherapy is more likely to be repeated in these patients in later cycles of chemotherapy. Patients with a shorter duration of nausea/vomiting are less likely to have repeat episodes of nausea/vomiting. The duration of chemotherapy induced nausea/vomiting should be considered for the prevention of future nausea/vomiting.

## Introduction

Chemotherapy-induced nausea and vomiting (CINV) are among the chemotherapy toxicities most feared by patients, impacting the quality and cost of cancer care. Although significant progress has been made in CINV prophylaxis, clinician adherence to evidence-based national and international antiemetic guidelines remains poor.^1-3^ Furthermore, even in clinical trials with protocol-required 100% triple prophylaxis (NK1 receptor antagonist [RA], 5-HT3 RA, dexamethasone [DEX]), CINV can occur in 10%-50% of patients receiving highly emetogenic chemotherapy.^4,5^ The continued high prevalence of CINV, involved in 10% of all avoidable acute care (ie, unplanned hospitalization or emergency department use) for Medicare patients receiving chemotherapy,^6^ led to its inclusion within Medicare’s sole medical oncology outcome quality measure, now publicly reported for each US hospital and directly driving total oncology reimbursement improvements or penalties.^7^

Prior CINV (during previous lines and cycles of therapy) is an established patient-specific risk factor for subsequent CINV, along with female sex and younger age.^8^ However, to our knowledge, no study has evaluated whether CINV’s duration is associated with its recurrence in subsequent cycles. Therefore, we performed a secondary analysis of a phase III, multicenter randomized trial (NCT03403712) in women with breast cancer naïve to highly or moderately emetogenic chemotherapy, which assessed the safety of oral vs. intravenous (IV) netupitant and palonosetron (NEPA) both combined with oral dexamethasone as antiemetic prophylaxis prior to ­anthracycline-cyclophosphamide (AC)-based chemotherapy for up to 4 cycles. This study established the efficacy and safety of IV NEPA in patients receiving AC. The objectives of this secondary analysis were to assess the combined (IV and oral NEPA) rate of complete response (CR, no nausea/vomiting or rescue antiemetic use) in the first cycle of chemotherapy (C1) and subsequent cycles (C2-4) and to evaluate the association between C1 CINV duration of subsequent treatment failure (TF; short vs. extended TF) and risk of subsequent CINV.

## Methods

### Study Sample

This secondary analysis included women with breast cancer treated with a single dose of oral NEPA (netupitant 300 mg and palonosetron 0.5 mg) or a single dose of IV NEPA (fosnetupitant 235 mg and palonosetron 0.25 mg), both combined with a single oral dose of dexamethasone (12 mg) as antiemetic prophylaxis before AC-based chemotherapy in a phase III randomized clinical trial. All participants gave their informed consent to participate in the study and the study was conducted in accordance with the provisions of the Declaration of Helsinki and with the approval of an Institutional Review Board.

This trial’s design, inclusion and exclusion criteria, CONSORT diagram, and results of the original trial have been published previously.^9^ No differences in ­treatment-emergent adverse events or efficacy were found between IV and oral NEPA in the trial. The present analysis pools all 402 patients treated with oral or IV NEPA.

### Outcomes

We hypothesized that, while the overall population CINV rates may be consistent across cycles within a clinical trial, individual patients experiencing CINV in C1 would experience a higher risk in each later cycle and that extended duration (≥3 days) of CINV would pose a greater risk of subsequent recurrence than short CINV (1-2 days). The duration of treatment failure (TF) was included as a prespecified secondary analysis in the statistical analysis plan dedicated to Health Economics and Outcomes Research (HEOR). We evaluated these factors as a secondary analysis to characterize the relationship between CINV duration and subsequent cycle treatment failure risk.

CR to antiemetic prophylaxis was defined as no emetic events or rescue medication use assessed daily over 5 days after receiving NEPA-based CINV prophylaxis. TF was defined as one or more emetic events or the need for rescue antiemetics assessed daily over 5 days. Patients with TF in C1 were further categorized into short TF (1-2 days of emetic events or rescue medication use within the 5 days) or extended TF (≥ 3 days of emetic events or rescue medication use within the 5 days). Assessment of efficacy was based on patients’ diaries, which captured emetic episodes and use of rescue medications daily from days 1 to 5 of each chemotherapy cycle. For each cycle, patients were classified as having a CR or TF.

### Statistical Analysis

Descriptive statistics of baseline patient characteristics were compared using *t*-tests or chi-square as appropriate. The proportion of patients meeting each category of CR and TF is reported for cycles 2-4.

To examine the relationship between first cycle response and subsequent cycle response (primary outcome measure), patients were first categorized as CR or TF based on their first cycle (CR1 or TF1, respectively). Then the percentage of subsequent cycles with either CR or TF are reported for each C1 response group. The proportion of patients achieving either CR or TF in any subsequent cycle was compared using a chi-square test.

To assess the effect of C1 TF duration on subsequent TF (secondary outcome measure), patients were categorized into short TF or extended TF based on their first cycle. The relative risk of TF for each category was calculated. Using generalized estimating equations (GEE), a multinomial logistic regression clustered on the patient was performed to estimate the probability of having CR, short TF, or extended TF in each subsequent cycle.

Analyses were done using SAS version 9.4 software and Stata MP version 15.0.

## Results

### Patient Characteristics

A total of 402 patients with breast cancer were treated with AC and received antiemetic prophylaxis with NEPA + DEX. Patient demographics stratified by CR1 or TF1 status and by short-TF1 or extended-TF1 status are summarized in Table 1. There were no demographic differences between patients experiencing short vs. extended TF1.

**Table 1.** Patient demographics for complete responders and treatment failures, and treatment failure duration.

	CR_1_ (*N* = 303)	TF_1_ (*N* = 99)	*P*-value	sTF_1_ (1-2 days) (*N* = 48)	xTF_1_ (≥3 days) (*N* = 51)	*P*-value
**Age (years)**						
Mean (SD)	56.3 (9.7)	52.5 (9.8)	.0007	53.4 (10.5)	51.6 (9.1)	.3538
Median (Q1-Q3)	57.0 (50.0-63.0)	53.0 (47.0-59.0)	.0006	52.5 (47.0-61.6)	53.0 (47.0-58.0)	.5968
Min, Max	27.0, 80.0	28.0, 81.0	—	34.0, 81.0	28.0, 69.0	—
Age group			.0541			.0960
18-49 years	74 (24%)	33 (33%)		16 (33%)	17 (33%)	
50-64 years	171 (56%)	56 (57%)		24 (50%)	32 (63%)	
65+ years	58 (19%)	10 (10%)		8 (17%)	2 (4%)	
Country, *n* (%)			<.0001			.2059
USA	36 (12%)	46 (46%)		20 (42%)	26 (51%)	
Georgia	46 (15%)	14 (14%)		5 (10%)	9 (18%)	
Ukraine	157 (52%)	24 (24%)		16 (33%)	8 (16%)	
Russia	64 (21%)	15 (15%)		7 (15%)	8 (16%)	
Race, *n* (%)			.3121			.5911
White	287 (95%)	88 (89%)		44 (92%)	44 (86%)	
Black or African American	7 (2%)	6 (6%)		3 (6%)	3 (6%)	
American Indian or Alaska Native	1 (0%)	0 (0%)		0 (0%)	0 (0%)	
Asian	1 (0%)	1 (1%)		0 (0%)	1 (2%)	
Native Hawaiian or Other Pacific Islander	1 (0%)	0 (0%)		0 (0%)	0 (0%)	
Other	1 (0%)	0 (0%)		0 (0%)	0 (0%)	
Unknown	5 (2%)	4 (4%)		1 (2%)	3 (6%)	

### Cycle to Cycle Response Rates

**Figure 1.**
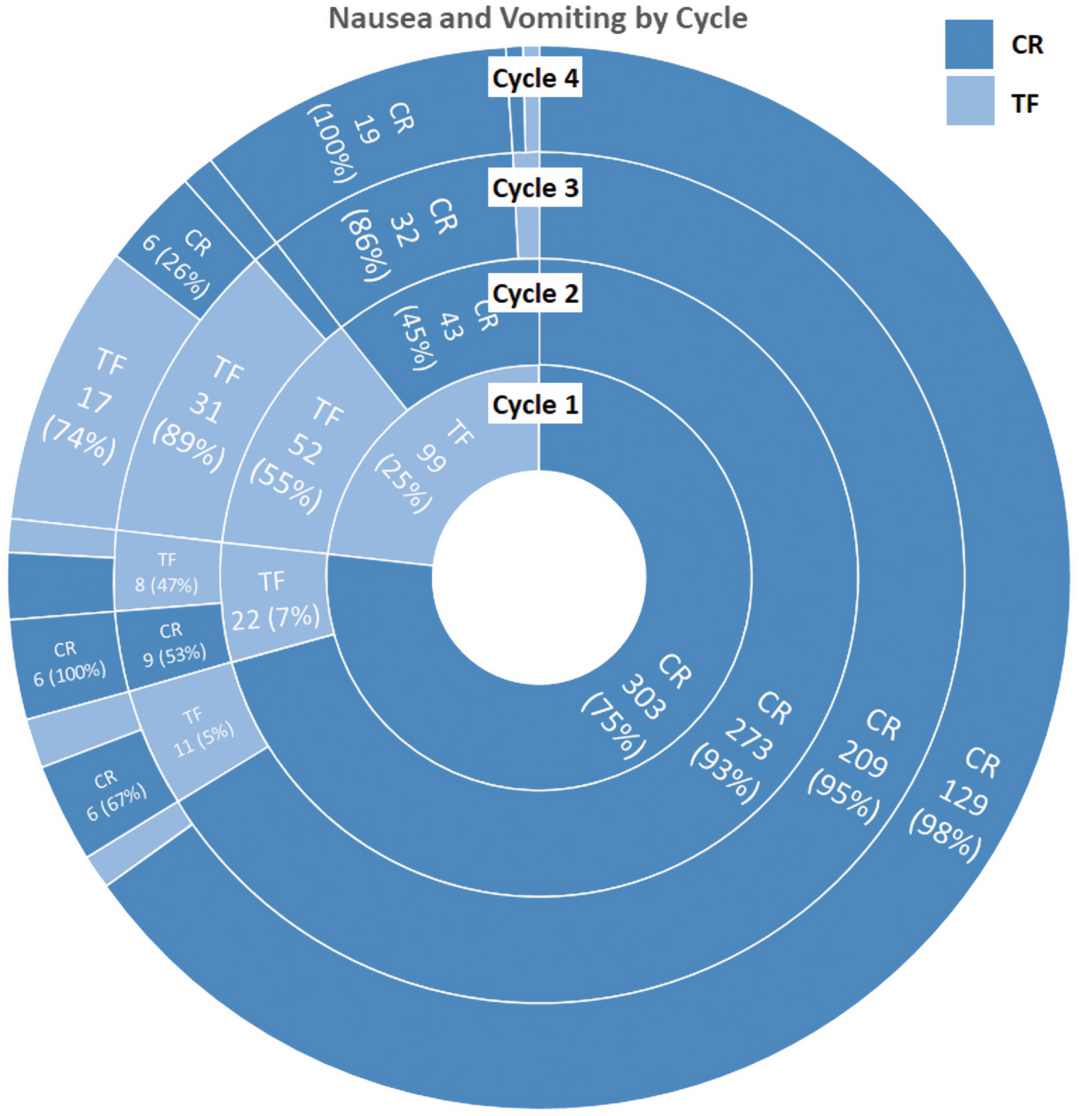
NEPA results of CR and TF for each cycle^a^. CR: complete response; TF: treatment failure. ^a^Note on interpreting this figure: Each ring represents a cycle and is partitioned by CR and TF. The subsequent ring is again partitioned by CR and TF and shows the proportion of patients with CR or TF in the subsequent cycle based on their status in the previous cycle. For example, in C1, 25% of patients were TF and 75% were CR. In C2, 55% of the 25% TF1 patients were TF again and 93% of the 75% CR1 patients were CR again.

Of the 402 patients, there were 303 (75.4%) patients with CR and 99 (24.6%) with TF (Fig. 1) in C1. Among the 303 patients with CR in C1, the following cycle CR rates were: 93% repeat CR in C2, 95% in C3, and 98% in C4. Conversely, among the 99 patients with TF in C1: 55% repeated TF in C2, 89% in C3, and 74% in C4 (Fig. 1).

In total, of 1299 cycles of AC treatment with NEPA + DEX prophylaxis, there were 1046 with CR and 253 with TF. While CR rates consistently averaged near 75% for the total population in each cycle, patients with CR in C1 had subsequent CR in 636/684 (93.0%) of C2-4, whereas patients with TF in C1 experienced TF in 106/213 (49.8%) of C2-4 (*P* < .0001).

### Treatment Failure Duration and Subsequent Cycle Failure

In the first cycle of prophylactic treatment with NEPA + DEX, 99 patients experienced TF (TF1). Of those, 48 (48.5%) were classified as short TF1 and 51 (51.5%) as extended TF1 (Table 1). For patients with short TF1 that continued, 32/46 (69.6%) subsequently achieved CR in C2 (2 patients discontinued treatment). Of those, 24/28 (86%) repeated CR in C3 (4 patients discontinued treatment), and 15/15 (100%) repeated CR in C4 (9 patients discontinued treatment). In contrast, for patients with extended TF1 continuing treatment, 38/49 (77.6%) experienced repeat TF in C2 (2 patients discontinued), 22/25 (88.0%) in C3 (13 patients discontinued), and 12/16 (75%) in C4 (6 patients discontinued).

Overall, extended TF1 resulted in CINV recurring in 73/105 (69.5%) later cycles compared to short TF1 [CINV recurring in 33/108 (30.6%) of later cycles; *P* < .001]. The absolute risk increase from extended TF was 40.4% (number needed to harm = 2), with a relative risk of recurrence of 2.28 (CI 1.67-3.11; *P* < .001). Individual patient’s duration of recurrence was consistent across cycles (*P* = .046). As a predictor of later cycle TF, extended TF had a positive predictive value of 70%, sensitivity of 69%, and specificity of 70%.

**Figure 2.**
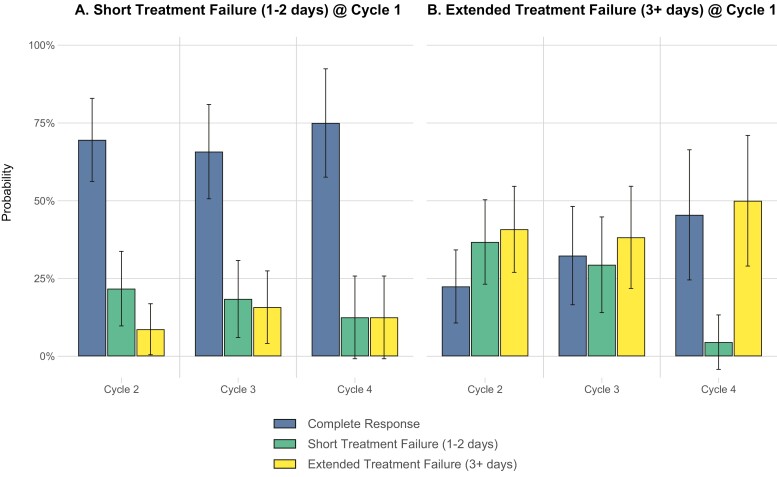
Probability of CR, short TF, or extended TF in subsequent cycles based on initial cycle classification.

The results of the GEE model showed that patients classified as short TF1 had a 69.6% (95% CI: 56.2%-82.9%) probability of achieving CR in C2 compared with a 22.5% (95% CI: 10.7%-34.2%) probability of patients classified as extended TF1 (*P* < .001). In subsequent cycles, the probability of achieving CR for short TF1 and extended TF1 patients in C3 was 65.8% (95% CI: 50.6%-81.0%) and 32.4% (95% CI: 16.5%-48.2%), respectively (*P* = .003) and 75% (95% CI: 57.6%-92.4%) compared with 45.5% (95% CI: 24.5%-66.4%), respectively, in C4 (*P* = .033) (Fig. 2; Supplementary Table S1).

## Discussion

We present the first data evaluating the duration of CINV in C1 as a predictor of subsequent CINV. While most patients achieved a CR, one-quarter of patients receiving AC did not, and of these, the patients with extended TF1 had >70% risk of subsequent CINV. One of the top 5 major cancer advances in the last 50 years has been the development of effective CINV prophylaxis.^10^ Our findings align with multiple analyses of large US databases which show that CINV occurs in over 25% of patients receiving highly emetogenic chemotherapy.^2,3^ The impact on these patients and the associated economic toll is considerable: 28% of patients receiving highly emetogenic chemotherapy require hospitalization or emergency department treatment within 30 days, including 21%-28% (for carboplatin and cisplatin, respectively) involved CINV.^1^

Our evaluation of CINV duration stemmed from our observation that the traditional CINV endpoint—any vomiting or use of rescue medication over 5 days ­post-chemotherapy—fails to distinguish mild/transient CINV from continuous/severe CINV, a distinction reflected in many validated measures of symptoms and toxicities, including the NCI’s PRO-CTCAE.^11^ This observation led us to explore whether extended duration CINV had a differential effect on subsequent CINV outcomes. Ballatori^12^ found CINV severity and, to a greater degree, CINV duration impact patient quality of life, while Roeland^13^ demonstrated that nausea severity was consistently low for short CINV and consistently higher for extended CINV. Together, these supported our bifurcation of TF duration into 1-2 days and 3-5 days. The results show patients with extended CINV faced >70% risk of later CINV, an absolute increase in the risk of >40% compared with short CINV (number needed to harm = 2). Further, the duration of subsequent CINV was typically consistent with the initial duration, meaning that those with extended CINV thereafter usually continued to have extended CINV in each later cycle—clearly, an unacceptable outcome. Even patients without extended CINV in early cycles shifted to ongoing extended CINV after a single extended CINV episode. Future clinical trials of antiemetic agents may benefit from monitoring CINV duration and individual patient CINV recurrence.

In clinical practice, we see considerable heterogeneity in the duration of CINV. Our findings show that CINV duration (short vs. extended) in C1 strongly predicts CINV recurrence in subsequent cycles. This higher risk from extended CINV is both statistically significant and clinically meaningful due to the longer duration and risk-prediction associated with extended TF and the work loss and impaired activity in this same study population.^14^ The high rates of avoidable acute care due to CINV are often difficult to document as linked to CINV duration since daily diaries used in clinical trials are lacking in real-world practice, while conversely, acute-care use is typically not prospectively tracked in clinical trials. Given the risk of acute-care use, clinicians should note any extended duration CINV during C1 and consider the potential advantage to modifying antiemetic prophylaxis in subsequent cycles.

Past trials have primarily reported consistent ­population-wide CINV rates across each successive cycle of HEC chemotherapy and may therefore mask an actual wide disparity between 2 distinct populations: a majority of patients with successful antiemetic prophylaxis who initially avoid CINV and subsequently have rare events, and a minority whose prophylaxis fails initially and, if of extended duration, persists throughout their therapy. Previous work by Schwartzberg et al.^15^ and Molasiotis et al.^8^ showed patients with CINV in C1 have an excess risk for later CINV without assessing the timing or frequency of recurrence or its association with duration.

The strengths of this report include the prespecified secondary analysis of CINV duration within a large, randomized, multicenter, phase III trial of a high-risk CINV patient population (ie, women receiving AC). A principal limitation is that this study involved a single chemotherapy regimen and a single antiemetic regimen. Additionally, nausea, which is correlated with vomiting, is not directly measured as part of the CR criteria used in this study. The differential impact of major CINV risk factors (eg, sex, age) was not examined due to the trial’s focus on AC treatment for breast cancer. Other limitations include limited racial diversity in the trial population and potential differences in treatment failure by geography. Future studies could characterize whether these findings apply to other high- or moderately emetogenic chemotherapy regimens and with different CINV prophylaxis.

Traditional descriptions of CINV have focused on acute CINV in the first 24 hours post-chemotherapy and delayed CINV in the 24-120 hours after chemotherapy, with acute CINV well controlled with current antiemetics while delayed CINV has been much less well controlled. Short and extended CINV appear to be more aligned with severity and may better predict patients’ experiences in later chemotherapy cycles. The finding that CINV duration in C1 of AC-based chemotherapy powerfully predicts CINV outcomes for the remainder of the patient’s chemotherapy, with an 80% risk of repeat failure after extended TF, provides evidence that CINV’s duration is a new and strong risk factor for recurrence that clinicians should consider in CINV prophylaxis treatment decisions. Extended CINV may be a considerably greater risk factor for future outcomes than all other known CINV risk factors combined.^8,14^ Future research should focus on those with extended TF to identify alternative prophylaxis strategies to reduce CINV in subsequent cycles, including investigating a guideline-compliant 4-component prophylaxis regimen or other augmented approaches.

## Conclusions

Consistent CR rates across cycles are commonly reported in trials of guideline-recommended triple antiemetic prophylaxis. Yet, these results likely mask marked differences in ­later-cycle CINV risks between patients with and without CINV in C1. We found prophylaxis success in C1 of AC-based chemotherapy led to high success rates across subsequent cycles, while conversely, C1 prophylaxis failure (ie, CINV) led to repeat failure in later cycles. Given the clinical impact of extended CINV, clinicians should monitor breakthrough CINV duration and consider optimizing antiemetic prophylaxis when extended CINV does occur.

## Supplementary Material

oyac240_suppl_Supplementary_Table_S1

## Data Availability

Requests for the clinical trial data can be directed to datasharing@helsinn.com.
