# Supplementary material for: Duration of Chemotherapy-induced Nausea and Vomiting (CINV) as a Predictor of Recurrent CINV in Later Cycles
Source: Oncologist. 2022 Dec 17;28(3):208–13. doi: 10.1093/oncolo/oyac240 (PMC10020801; doi:10.1093/oncolo/oyac240)
Supplement: oyac240_suppl_Supplementary_Table_S1 [file oyac240_suppl_supplementary_table_s1.docx]

**Supplemental Table 1.** Probability of CR, short TF, or extended TF in subsequent cycles based on initial cycle classification

|  | **At Cycle 1** | | | | | |
| --- | --- | --- | --- | --- | --- | --- |
|  | **Short Treatment Failure  (1-2 days)** | | **Extended Treatment Failure  (3+ days)** | | **Extended vs. Short** | |
|  | **Predicted Probability** | **95% CI** | **Predicted Probability** | **95% CI** | \| **Predicted Probability** \| \| --- \| | ***p*** |
|  |  |  |  |  |  |  |
| **At Cycle 2** |  |  |  |  |  |  |
| Complete Response | 69.6% | (56.20, 82.93) | 22.5% | (10.70, 34.19) | -47.1% | <0.001 |
| Short Treatment Failure | 21.7% | (9.76, 33.72) | 36.7% | (23.17, 50.30) | 15.0% | 0.104 |
| Extended Treatment Failure | 8.7% | (0.51, 16.88) | 40.8% | (26.98, 54.65) | \| 32.1% \| \| --- \| | <0.001 |
|  |  |  |  |  |  |  |
| **At Cycle 3** |  |  |  |  |  |  |
| Complete Response | 65.8% | (50.63, 80.95) | 32.4% | (16.54, 48.16) | -33.4% | 0.003 |
| Short Treatment Failure | 18.4% | (6.03, 30.81) | 29.4% | (14.01, 44.81) | 11.0% | 0.276 |
| Extended Treatment Failure | 15.8% | (4.13, 27.44) | 38.2% | (21.81, 54.66) | 22.5% | 0.029 |
|  |  |  |  |  |  |  |
| **At Cycle 4** |  |  |  |  |  |  |
| Complete Response | 75.0% | (57.58, 92.42) | 45.5% | (24.54, 66.37) | -29.6% | 0.033 |
| Short Treatment Failure | 12.5% | (-0.80, 25.80) | 4.6% | (-4.20, 13.30) | -8.0% | 0.327 |
| Extended Treatment Failure | 12.5% | (-0.80, 25.80) | 50.0% | (29.00, 71.00) | 37.5% | 0.003 |
|  |  |  |  |  |  |  |
